# Dimethyl 2-[22,24-dimethyl-23-oxo-8,11,14-trioxa-25-aza­tetra­cyclo­[19.3.1.0^2,7^.0^15,20^]penta­cosa-2,4,6,15(20),16,18-hexaen-25-yl]but-2-enedioate

**DOI:** 10.1107/S1600536812018867

**Published:** 2012-05-02

**Authors:** Le Tuan Anh, Truong Hong Hieu, Anatoly T. Soldatenkov, Nadezhda M. Kolyadina, Victor N. Khrustalev

**Affiliations:** a Department of Chemistry, Vietnam National University, 144 Xuan Thuy, Cau Giay, Hanoi, Vietnam; b Organic Chemistry Department, Russian Peoples Friendship University, Miklukho-Maklaya St 6, Moscow 117198, Russian Federation; c X-Ray Structural Centre, A.N. Nesmeyanov Institute of Organoelement Compounds, Russian Academy of Sciences, 28 Vavilov St, B-334, Moscow 119991, Russian Federation

## Abstract

The title compound, C_29_H_33_NO_8_, is a product of the Michael addition of the cyclic secondary amine subunit of the aza-14-crown-4 ether to dimethyl acetyl­enedicarboxyl­ate. The piperidinone ring exhibits a distorted chair conformation, and the dimethyl ethylenedicarboxylate fragment has a *cis* configuration with a dihedral angle of 78.96 (5)° between the two carboxyl­ate groups. The crystal packing is stabilized by weak C—H⋯O hydrogen bonds.

## Related literature

For general background to the design, synthesis, chemical properties and applications of macrocyclic ligands for coordination chemistry, see: Hiraoka (1978 ▶); Pedersen (1988 ▶); Schwan & Warkentin (1988 ▶); Gokel & Murillo (1996 ▶); Bradshaw & Izatt (1997 ▶). For related compounds, see: Levov *et al.* (2006 ▶, 2008 ▶); Anh *et al.* (2008 ▶, 2012 ▶); Hieu *et al.* (2011 ▶); Khieu *et al.* (2011 ▶).
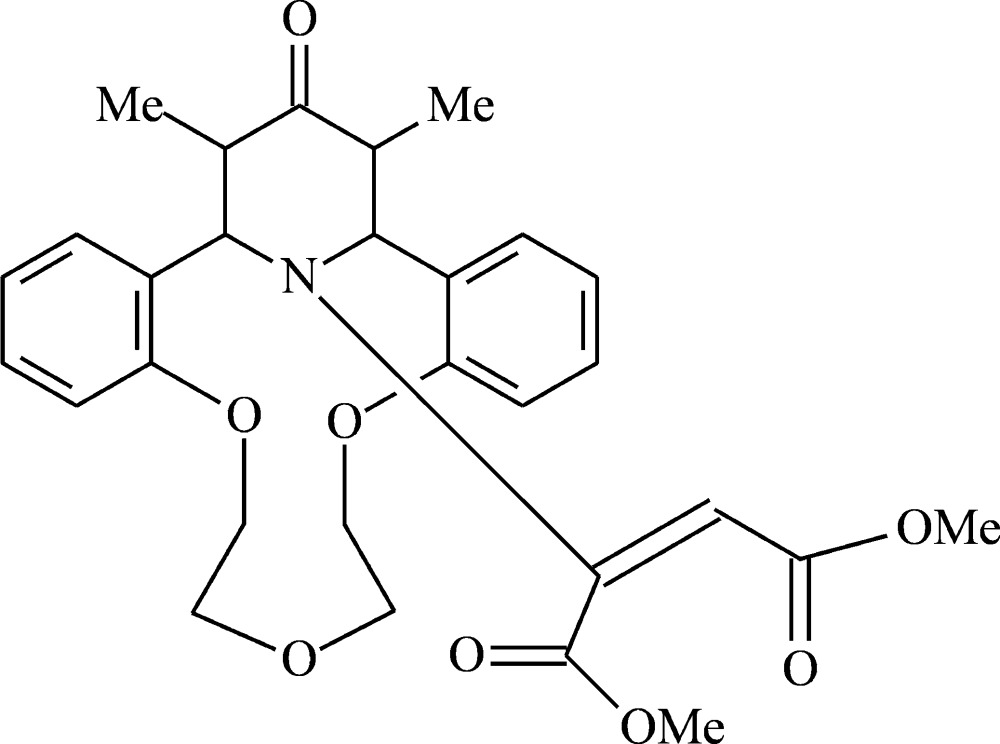


## Experimental

### 

#### Crystal data

C_29_H_33_NO_8_
*M* *_r_* = 523.56
Triclinic, 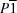

*a* = 8.8135 (4) Å
*b* = 8.9469 (4) Å
*c* = 18.3067 (9) Å
α = 79.077 (1)°
β = 78.218 (1)°
γ = 69.800 (1)°
*V* = 1315.05 (11) Å^3^
*Z* = 2
Mo *K*α radiation
μ = 0.10 mm^−1^
*T* = 100 K
0.30 × 0.25 × 0.25 mm

#### Data collection

Bruker APEXII CCD diffractometer
Absorption correction: multi-scan (*SADABS*; Sheldrick, 2003 ▶) *T*_min_ = 0.972, *T*_max_ = 0.976
17255 measured reflections
7669 independent reflections
6322 reflections with *I* > 2σ(*I*)
*R*_int_ = 0.024

#### Refinement

*R*[*F* ^2^ > 2σ(*F* ^2^)] = 0.044
*wR*(*F* ^2^) = 0.117
*S* = 1.00
7669 reflections
347 parameters
H-atom parameters constrained
Δρ_max_ = 0.48 e Å^−3^
Δρ_min_ = −0.26 e Å^−3^

### 

Data collection: *APEX2* (Bruker, 2005 ▶); cell refinement: *SAINT-Plus* (Bruker, 2001 ▶); data reduction: *SAINT-Plus*; program(s) used to solve structure: *SHELXTL* (Sheldrick, 2008 ▶); program(s) used to refine structure: *SHELXTL*; molecular graphics: *SHELXTL*; software used to prepare material for publication: *SHELXTL*.

## Supplementary Material

Crystal structure: contains datablock(s) global, I. DOI: 10.1107/S1600536812018867/cv5289sup1.cif

Structure factors: contains datablock(s) I. DOI: 10.1107/S1600536812018867/cv5289Isup2.hkl

Supplementary material file. DOI: 10.1107/S1600536812018867/cv5289Isup3.cml

Additional supplementary materials:  crystallographic information; 3D view; checkCIF report

## Figures and Tables

**Table 1:** Hydrogen-bond geometry (Å, °)

*D*—H⋯*A*	*D*—H	H⋯*A*	*D*⋯*A*	*D*—H⋯*A*
C13—H13*A*⋯O1^i^	0.99	2.56	3.2877 (18)	130
C29—H29*A*⋯O3^ii^	0.98	2.44	3.2498 (18)	139
C33—H33*A*⋯O5^iii^	0.98	2.56	3.4092 (16)	145

Symmetry codes: (i) 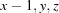; (ii) 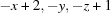; (iii) 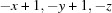.

**Fig. 1.**
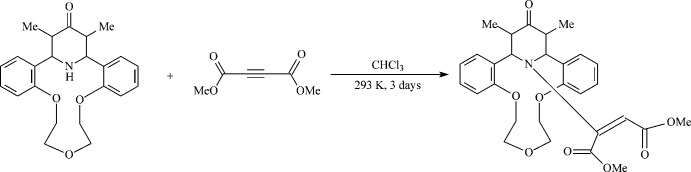
Michael addition of bis(benzo)-(β,β'-dimethyl-γ-piperidono)aza-14-crown-4 ether to dimethyl acetylenedicarboxylate.

**Fig. 2.**
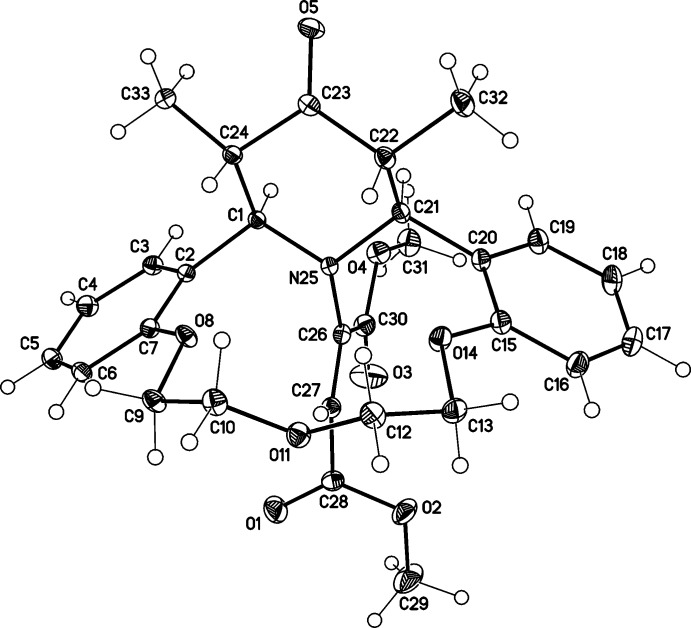
Molecular structure of I. Displacement ellipsoids are shown at the 50% probability level. H atoms are presented as small spheres of arbitrary radius.

## supplementary crystallographic information

### Comment

Design, synthesis and applications of macrocyclic ligands for coordination and supramolecular chemistry draw very great attention of investigators during the last forty years (Hiraoka, 1978; Pedersen, 1988; Gokel & Murillo, 1996; Bradshaw & Izatt, 1997). Recently we have developed the effective methods of synthesis of azacrown ethers containing piperidine (Levov *et al.*, 2006, 2008; Anh *et al.*, 2008, 2012), perhydropyrimidine (Hieu *et al.*, 2011) and perhydrotriazine subunits (Khieu *et al.*, 2011).

In attempts to apply this chemistry for obtaining of a macrocyclic ligand bringing the desirable functional groups, we studied the Michael addition of the cyclic secondary amine subunit of the crown ether to dimethyl ethylenedicarboxylate. The expected reaction is well known (Schwan & Warkentin, 1988), but might be highly hindered due to the steric reasons. We have found, however, that the expected *N*-vynilation proceeded smoothly with the formation of an *N*-maleinate derivative of the azacrown system.

The title compound I, C_29_H_33_NO_8_, is a product of the Michael addition of the cyclic secondary amine subunit of the aza-14-crown-4 ether to dimethyl acetylenedicarboxylate (Figure 1). The title macromolecule includes the aza-14-crown-4-ether skeletal moiety and adopts a bowl conformation (Figure 2). The configuration of the C7—O8—C9—C10—O11—C12—C13—O14—C15 polyether chain is t–g^(-)^–t–t–g^(+)^–t (t = *trans*, 180°; g = *gauche*, ±60°). The piperidinone ring of the bicyclic fragment have a slightly flattenned *chair* conformation. The dihedral angle between the planes of the benzene rings fused to the aza-14-crown-4-ether moiety is 56.33 (4)°. The methyl substituents at the C22 and C24 carbon atoms occupy the sterically favorable equatorial positions. The carboxylate substituents are rotated to each other by 78.96 (5)°. The volume of the internal cavity of macrocycle I is approximately equal to 57 Å^3^.

The molecule of I possesses four asymmetric centers at the C1, C21, C22 and C24 carbon atoms and can have potentially numerous diastereomers. The crystal of I is racemic and consists of enantiomeric pairs with the following relative configuration of the centers: *rac*-1*R**,21*S**,22*R**,24*S**.

In the crystal, the molecules of I are bound to each other by weak C—H···O hydrogen bonding interactions (Table 1) into three-dimensional framework.

### Experimental

Dimethyl acetylenedicarboxylate (0.11 g, 0.79 mmol) was added to a solution of bis(benzo)-(β,β'-dimethyl-γ-piperidono)aza-14-crown-4 ether (0.30 g, 0.79 mmol) in chloroform (20 ml). The reaction mixture was stirred at 293 K for 3 days (monitoring by TLC until disappearance of the starting organic compounds spots). At the end of the reaction, the formed precipitate was separated, washed with cold chloroform (40 ml) and re-crystallized from ethanol to give 0.41 g of pale yellow crystals of I. Yield is 99%. *M*.p. = 506–508 K. IR (KBr), ν/cm^-1^: 1599, 1643, 1710, 1729. ^1^H NMR (CDCl_3_, 400 MHz, 300 K): δ = 0.43 (d, 6H, C—CH_3_, *J* = 7.0), 3.00 and 3.17 (both s, 3H each, CH_3_), 3.46, 3.54, 3.60 and 3.68 (all m, 2H, 4H, 2H and 2H, respectively, H22, H24 and OCH_2_CH_2_OCH_2_CH_2_O), 3.76 and 3.83 (both d, 1H each, H1 and H21, *J* = 10.1), 6.39 and 6.43 (both m, 2H each, H_arom_), 6.49(c, 1H, O_2_C—CH=C—CO_2_), 6.58 (dd, 2H, *J* = 7.2 and 2.0), 6.86 (tt, 2H, *J* = 8.4 and 2.0, H_arom_). Anal. Calcd for C_29_H_33_NO_8_: C, 66.53; H, 6.35; N, 2.68. Found: C, 66.81; H, 6.70; N, 2.75.

### Refinement

The hydrogen atoms were placed in calculated positions with C—H = 0.95–1.00 Å and refined in the riding model with fixed isotropic displacement parameters [*U*_iso_(H) = 1.5*U*_eq_(C) for the methyl groups and 1.2*U*_eq_(C) for the other groups].

### Crystal data

**Table:**

C_29_H_33_NO_8_	*Z* = 2
*M**_r_* = 523.56	*F*(000) = 556
Triclinic, *P*1	*D*_x_ = 1.322 Mg m^−^^3^
Hall symbol: -P 1	Mo *K*α radiation, λ = 0.71073 Å
*a* = 8.8135 (4) Å	Cell parameters from 7696 reflections
*b* = 8.9469 (4) Å	θ = 2.3–32.6°
*c* = 18.3067 (9) Å	µ = 0.10 mm^−^^1^
α = 79.077 (1)°	*T* = 100 K
β = 78.218 (1)°	Prism, yellow
γ = 69.800 (1)°	0.30 × 0.25 × 0.25 mm
*V* = 1315.05 (11) Å^3^	

### Data collection

**Table:**

Bruker APEXII CCD diffractometer	7669 independent reflections
Radiation source: fine-focus sealed tube	6322 reflections with *I* > 2σ(*I*)
Graphite monochromator	*R*_int_ = 0.024
φ and ω scans	θ_max_ = 30.0°, θ_min_ = 2.3°
Absorption correction: multi-scan (*SADABS*; Sheldrick, 2003)	*h* = −12→12
*T*_min_ = 0.972, *T*_max_ = 0.976	*k* = −12→12
17255 measured reflections	*l* = −25→25

### Refinement

**Table:**

Refinement on *F*^2^	Primary atom site location: structure-invariant direct methods
Least-squares matrix: full	Secondary atom site location: difference Fourier map
*R*[*F*^2^ > 2σ(*F*^2^)] = 0.044	Hydrogen site location: inferred from neighbouring sites
*wR*(*F*^2^) = 0.117	H-atom parameters constrained
*S* = 1.00	*w* = 1/[σ^2^(*F*_o_^2^) + (0.0585*P*)^2^ + 0.545*P*] where *P* = (*F*_o_^2^ + 2*F*_c_^2^)/3
7669 reflections	(Δ/σ)_max_ < 0.001
347 parameters	Δρ_max_ = 0.48 e Å^−^^3^
0 restraints	Δρ_min_ = −0.26 e Å^−^^3^

### Special details

**Table:**

Geometry. All e.s.d.'s (except the e.s.d. in the dihedral angle between two l.s. planes) are estimated using the full covariance matrix. The cell e.s.d.'s are taken into account individually in the estimation of e.s.d.'s in distances, angles and torsion angles; correlations between e.s.d.'s in cell parameters are only used when they are defined by crystal symmetry. An approximate (isotropic) treatment of cell e.s.d.'s is used for estimating e.s.d.'s involving l.s. planes.
Refinement. Refinement of *F*^2^ against ALL reflections. The weighted *R*-factor *wR* and goodness of fit *S* are based on *F*^2^, conventional *R*-factors *R* are based on *F*, with *F* set to zero for negative *F*^2^. The threshold expression of *F*^2^ > σ(*F*^2^) is used only for calculating *R*-factors(gt) *etc*. and is not relevant to the choice of reflections for refinement. *R*-factors based on *F*^2^ are statistically about twice as large as those based on *F*, and *R*- factors based on ALL data will be even larger.

### Fractional atomic coordinates and isotropic or equivalent isotropic displacement parameters (Å^2^)

**Table:**

	*x*	*y*	*z*	*U*_iso_*/*U*_eq_	
O1	1.13161 (11)	−0.16633 (12)	0.31814 (5)	0.0276 (2)	
O2	0.92344 (12)	−0.11393 (13)	0.41308 (5)	0.0310 (2)	
O3	1.00781 (15)	0.19034 (13)	0.35751 (6)	0.0359 (3)	
O4	0.85150 (11)	0.41619 (10)	0.29707 (5)	0.01918 (17)	
O5	0.47470 (11)	0.60218 (10)	0.08211 (5)	0.02019 (18)	
C1	0.81920 (13)	0.31443 (12)	0.14927 (6)	0.01163 (19)	
H1	0.8403	0.4119	0.1582	0.014*	
C2	0.98327 (13)	0.19396 (13)	0.12362 (6)	0.01201 (19)	
C3	1.12344 (13)	0.23975 (13)	0.10998 (6)	0.0146 (2)	
H3	1.1152	0.3423	0.1208	0.018*	
C4	1.27594 (14)	0.13916 (14)	0.08085 (7)	0.0172 (2)	
H4	1.3694	0.1739	0.0709	0.021*	
C5	1.28898 (14)	−0.01176 (14)	0.06661 (6)	0.0168 (2)	
H5	1.3922	−0.0814	0.0472	0.020*	
C6	1.15144 (13)	−0.06216 (13)	0.08057 (6)	0.0153 (2)	
H6	1.1615	−0.1663	0.0713	0.018*	
C7	0.99921 (13)	0.04013 (13)	0.10818 (6)	0.01280 (19)	
O8	0.85858 (9)	0.00052 (9)	0.12206 (5)	0.01476 (16)	
C9	0.86878 (14)	−0.15809 (13)	0.11310 (7)	0.0161 (2)	
H9A	0.9469	−0.2391	0.1441	0.019*	
H9B	0.9060	−0.1764	0.0598	0.019*	
C10	0.69933 (14)	−0.16997 (14)	0.13859 (7)	0.0176 (2)	
H10A	0.6193	−0.0781	0.1131	0.021*	
H10B	0.6944	−0.2704	0.1254	0.021*	
O11	0.66061 (10)	−0.16906 (10)	0.21772 (5)	0.01780 (17)	
C12	0.48952 (14)	−0.13173 (14)	0.24487 (7)	0.0183 (2)	
H12A	0.4585	−0.2308	0.2550	0.022*	
H12B	0.4261	−0.0563	0.2066	0.022*	
C13	0.45236 (15)	−0.05669 (14)	0.31593 (7)	0.0190 (2)	
H13A	0.3363	−0.0381	0.3384	0.023*	
H13B	0.5221	−0.1275	0.3531	0.023*	
O14	0.48591 (11)	0.09270 (10)	0.29488 (5)	0.01821 (17)	
C15	0.46024 (14)	0.18691 (14)	0.34945 (6)	0.0164 (2)	
C16	0.39367 (15)	0.15084 (15)	0.42393 (7)	0.0212 (2)	
H16	0.3667	0.0544	0.4394	0.025*	
C17	0.36684 (16)	0.25667 (17)	0.47570 (7)	0.0240 (3)	
H17	0.3216	0.2319	0.5264	0.029*	
C18	0.40564 (16)	0.39754 (16)	0.45368 (7)	0.0233 (3)	
H18	0.3852	0.4706	0.4888	0.028*	
C19	0.47515 (15)	0.43101 (15)	0.37938 (7)	0.0191 (2)	
H19	0.5033	0.5269	0.3646	0.023*	
C20	0.50442 (13)	0.32768 (13)	0.32630 (6)	0.0153 (2)	
C21	0.57630 (13)	0.37356 (13)	0.24585 (6)	0.01339 (19)	
H21	0.5980	0.4769	0.2439	0.016*	
C22	0.45975 (13)	0.40020 (13)	0.18780 (6)	0.0150 (2)	
H22	0.4553	0.2934	0.1812	0.018*	
C23	0.53992 (13)	0.47026 (13)	0.11450 (6)	0.0138 (2)	
C24	0.71063 (13)	0.36643 (13)	0.08583 (6)	0.01280 (19)	
H24	0.7019	0.2674	0.0717	0.015*	
N25	0.73307 (11)	0.25314 (11)	0.22023 (5)	0.01215 (17)	
C26	0.83687 (13)	0.17022 (13)	0.27554 (6)	0.01333 (19)	
C27	0.87169 (13)	0.01127 (13)	0.29155 (6)	0.0148 (2)	
H27	0.8154	−0.0378	0.2695	0.018*	
C28	0.99175 (14)	−0.09596 (14)	0.34131 (6)	0.0165 (2)	
C29	1.0350 (2)	−0.2151 (2)	0.46417 (8)	0.0398 (4)	
H29A	0.9748	−0.2231	0.5153	0.060*	
H29B	1.1207	−0.1680	0.4634	0.060*	
H29C	1.0849	−0.3224	0.4484	0.060*	
C30	0.90969 (15)	0.25603 (14)	0.31496 (6)	0.0180 (2)	
C31	0.89526 (17)	0.51070 (16)	0.34025 (7)	0.0243 (3)	
H31A	0.8661	0.6228	0.3167	0.036*	
H31B	1.0132	0.4685	0.3416	0.036*	
H31C	0.8361	0.5049	0.3917	0.036*	
C32	0.28641 (15)	0.50733 (16)	0.21208 (7)	0.0231 (2)	
H32A	0.2222	0.5306	0.1711	0.035*	
H32B	0.2901	0.6080	0.2242	0.035*	
H32C	0.2355	0.4522	0.2566	0.035*	
C33	0.78760 (14)	0.45247 (14)	0.01605 (6)	0.0161 (2)	
H33A	0.7183	0.4816	−0.0234	0.024*	
H33B	0.8962	0.3813	−0.0020	0.024*	
H33C	0.7979	0.5499	0.0286	0.024*	

### Atomic displacement parameters (Å^2^)

**Table:**

	*U*^11^	*U*^22^	*U*^33^	*U*^12^	*U*^13^	*U*^23^
O1	0.0192 (4)	0.0318 (5)	0.0246 (5)	0.0014 (4)	−0.0035 (3)	−0.0044 (4)
O2	0.0206 (5)	0.0405 (6)	0.0195 (4)	−0.0025 (4)	−0.0026 (3)	0.0111 (4)
O3	0.0498 (7)	0.0263 (5)	0.0397 (6)	−0.0101 (5)	−0.0305 (5)	−0.0021 (4)
O4	0.0235 (4)	0.0171 (4)	0.0210 (4)	−0.0094 (3)	−0.0050 (3)	−0.0045 (3)
O5	0.0182 (4)	0.0139 (4)	0.0251 (4)	−0.0013 (3)	−0.0047 (3)	−0.0001 (3)
C1	0.0116 (4)	0.0106 (4)	0.0123 (4)	−0.0034 (4)	−0.0010 (3)	−0.0018 (3)
C2	0.0114 (4)	0.0120 (5)	0.0117 (4)	−0.0028 (4)	−0.0017 (3)	−0.0011 (3)
C3	0.0144 (5)	0.0140 (5)	0.0162 (5)	−0.0058 (4)	−0.0024 (4)	−0.0010 (4)
C4	0.0123 (5)	0.0181 (5)	0.0206 (5)	−0.0052 (4)	−0.0018 (4)	−0.0011 (4)
C5	0.0115 (5)	0.0176 (5)	0.0186 (5)	−0.0020 (4)	−0.0012 (4)	−0.0024 (4)
C6	0.0144 (5)	0.0126 (5)	0.0170 (5)	−0.0023 (4)	−0.0013 (4)	−0.0030 (4)
C7	0.0121 (5)	0.0128 (5)	0.0134 (5)	−0.0042 (4)	−0.0016 (4)	−0.0015 (4)
O8	0.0120 (4)	0.0109 (3)	0.0219 (4)	−0.0044 (3)	0.0003 (3)	−0.0052 (3)
C9	0.0161 (5)	0.0115 (5)	0.0220 (5)	−0.0048 (4)	−0.0016 (4)	−0.0052 (4)
C10	0.0185 (5)	0.0163 (5)	0.0212 (5)	−0.0084 (4)	−0.0015 (4)	−0.0061 (4)
O11	0.0150 (4)	0.0192 (4)	0.0191 (4)	−0.0060 (3)	−0.0012 (3)	−0.0026 (3)
C12	0.0155 (5)	0.0170 (5)	0.0239 (6)	−0.0081 (4)	0.0001 (4)	−0.0041 (4)
C13	0.0216 (6)	0.0161 (5)	0.0205 (5)	−0.0107 (4)	0.0010 (4)	−0.0012 (4)
O14	0.0242 (4)	0.0152 (4)	0.0174 (4)	−0.0111 (3)	0.0023 (3)	−0.0039 (3)
C15	0.0148 (5)	0.0171 (5)	0.0174 (5)	−0.0053 (4)	−0.0001 (4)	−0.0042 (4)
C16	0.0224 (6)	0.0226 (6)	0.0186 (5)	−0.0107 (5)	0.0021 (4)	−0.0019 (4)
C17	0.0237 (6)	0.0315 (7)	0.0160 (5)	−0.0106 (5)	0.0031 (4)	−0.0045 (5)
C18	0.0239 (6)	0.0285 (6)	0.0190 (6)	−0.0101 (5)	0.0036 (4)	−0.0108 (5)
C19	0.0186 (5)	0.0194 (5)	0.0201 (5)	−0.0072 (4)	0.0016 (4)	−0.0069 (4)
C20	0.0135 (5)	0.0165 (5)	0.0154 (5)	−0.0047 (4)	0.0005 (4)	−0.0037 (4)
C21	0.0125 (5)	0.0121 (5)	0.0151 (5)	−0.0040 (4)	0.0007 (4)	−0.0036 (4)
C22	0.0118 (5)	0.0151 (5)	0.0177 (5)	−0.0041 (4)	−0.0009 (4)	−0.0029 (4)
C23	0.0131 (5)	0.0128 (5)	0.0171 (5)	−0.0042 (4)	−0.0038 (4)	−0.0039 (4)
C24	0.0132 (5)	0.0115 (4)	0.0132 (5)	−0.0027 (4)	−0.0026 (4)	−0.0020 (3)
N25	0.0113 (4)	0.0119 (4)	0.0116 (4)	−0.0025 (3)	−0.0006 (3)	−0.0013 (3)
C26	0.0128 (5)	0.0155 (5)	0.0116 (4)	−0.0047 (4)	−0.0007 (4)	−0.0023 (4)
C27	0.0135 (5)	0.0160 (5)	0.0143 (5)	−0.0046 (4)	−0.0013 (4)	−0.0015 (4)
C28	0.0177 (5)	0.0153 (5)	0.0175 (5)	−0.0065 (4)	−0.0040 (4)	−0.0007 (4)
C29	0.0299 (7)	0.0530 (10)	0.0234 (7)	−0.0045 (7)	−0.0089 (6)	0.0145 (6)
C30	0.0213 (5)	0.0184 (5)	0.0161 (5)	−0.0074 (4)	−0.0036 (4)	−0.0033 (4)
C31	0.0294 (6)	0.0251 (6)	0.0251 (6)	−0.0148 (5)	−0.0011 (5)	−0.0111 (5)
C32	0.0130 (5)	0.0273 (6)	0.0247 (6)	−0.0018 (4)	−0.0006 (4)	−0.0046 (5)
C33	0.0164 (5)	0.0156 (5)	0.0143 (5)	−0.0037 (4)	−0.0020 (4)	−0.0005 (4)

### Geometric parameters (Å, º)

**Table:**

O1—C28	1.2031 (15)	C15—C16	1.3947 (16)
O2—C28	1.3335 (14)	C15—C20	1.4094 (16)
O2—C29	1.4495 (16)	C16—C17	1.3944 (18)
O3—C30	1.2045 (15)	C16—H16	0.9500
O4—C30	1.3442 (14)	C17—C18	1.3845 (19)
O4—C31	1.4461 (14)	C17—H17	0.9500
O5—C23	1.2167 (14)	C18—C19	1.3954 (17)
C1—N25	1.4761 (13)	C18—H18	0.9500
C1—C2	1.5203 (14)	C19—C20	1.3924 (16)
C1—C24	1.5564 (14)	C19—H19	0.9500
C1—H1	1.0000	C20—C21	1.5194 (15)
C2—C3	1.3932 (15)	C21—N25	1.4794 (13)
C2—C7	1.4113 (15)	C21—C22	1.5563 (15)
C3—C4	1.3981 (15)	C21—H21	1.0000
C3—H3	0.9500	C22—C23	1.5142 (15)
C4—C5	1.3851 (16)	C22—C32	1.5242 (16)
C4—H4	0.9500	C22—H22	1.0000
C5—C6	1.3948 (16)	C23—C24	1.5163 (15)
C5—H5	0.9500	C24—C33	1.5227 (15)
C6—C7	1.3940 (15)	C24—H24	1.0000
C6—H6	0.9500	N25—C26	1.4274 (13)
C7—O8	1.3664 (13)	C26—C27	1.3341 (15)
O8—C9	1.4292 (13)	C26—C30	1.5037 (16)
C9—C10	1.5052 (16)	C27—C28	1.4937 (15)
C9—H9A	0.9900	C27—H27	0.9500
C9—H9B	0.9900	C29—H29A	0.9800
C10—O11	1.4198 (14)	C29—H29B	0.9800
C10—H10A	0.9900	C29—H29C	0.9800
C10—H10B	0.9900	C31—H31A	0.9800
O11—C12	1.4288 (14)	C31—H31B	0.9800
C12—C13	1.5017 (17)	C31—H31C	0.9800
C12—H12A	0.9900	C32—H32A	0.9800
C12—H12B	0.9900	C32—H32B	0.9800
C13—O14	1.4331 (13)	C32—H32C	0.9800
C13—H13A	0.9900	C33—H33A	0.9800
C13—H13B	0.9900	C33—H33B	0.9800
O14—C15	1.3619 (14)	C33—H33C	0.9800
			
C28—O2—C29	114.81 (10)	C20—C19—H19	119.1
C30—O4—C31	115.93 (10)	C18—C19—H19	119.1
N25—C1—C2	112.76 (8)	C19—C20—C15	118.01 (10)
N25—C1—C24	110.82 (8)	C19—C20—C21	119.09 (10)
C2—C1—C24	109.93 (8)	C15—C20—C21	122.86 (10)
N25—C1—H1	107.7	N25—C21—C20	112.83 (9)
C2—C1—H1	107.7	N25—C21—C22	107.37 (8)
C24—C1—H1	107.7	C20—C21—C22	113.45 (9)
C3—C2—C7	117.97 (10)	N25—C21—H21	107.6
C3—C2—C1	119.08 (9)	C20—C21—H21	107.6
C7—C2—C1	122.83 (9)	C22—C21—H21	107.6
C2—C3—C4	121.90 (10)	C23—C22—C32	112.42 (9)
C2—C3—H3	119.0	C23—C22—C21	105.42 (9)
C4—C3—H3	119.0	C32—C22—C21	112.95 (9)
C5—C4—C3	119.14 (10)	C23—C22—H22	108.6
C5—C4—H4	120.4	C32—C22—H22	108.6
C3—C4—H4	120.4	C21—C22—H22	108.6
C4—C5—C6	120.41 (10)	O5—C23—C22	122.66 (10)
C4—C5—H5	119.8	O5—C23—C24	122.31 (10)
C6—C5—H5	119.8	C22—C23—C24	114.99 (9)
C7—C6—C5	120.08 (10)	C23—C24—C33	111.37 (9)
C7—C6—H6	120.0	C23—C24—C1	110.07 (9)
C5—C6—H6	120.0	C33—C24—C1	111.12 (9)
O8—C7—C6	123.53 (10)	C23—C24—H24	108.0
O8—C7—C2	115.99 (9)	C33—C24—H24	108.0
C6—C7—C2	120.48 (10)	C1—C24—H24	108.0
C7—O8—C9	118.60 (8)	C26—N25—C1	113.70 (8)
O8—C9—C10	106.33 (9)	C26—N25—C21	116.78 (8)
O8—C9—H9A	110.5	C1—N25—C21	112.17 (8)
C10—C9—H9A	110.5	C27—C26—N25	118.50 (10)
O8—C9—H9B	110.5	C27—C26—C30	119.42 (10)
C10—C9—H9B	110.5	N25—C26—C30	122.07 (10)
H9A—C9—H9B	108.7	C26—C27—C28	125.47 (10)
O11—C10—C9	108.85 (9)	C26—C27—H27	117.3
O11—C10—H10A	109.9	C28—C27—H27	117.3
C9—C10—H10A	109.9	O1—C28—O2	123.98 (11)
O11—C10—H10B	109.9	O1—C28—C27	123.42 (11)
C9—C10—H10B	109.9	O2—C28—C27	112.43 (10)
H10A—C10—H10B	108.3	O2—C29—H29A	109.5
C10—O11—C12	113.40 (9)	O2—C29—H29B	109.5
O11—C12—C13	108.73 (9)	H29A—C29—H29B	109.5
O11—C12—H12A	109.9	O2—C29—H29C	109.5
C13—C12—H12A	109.9	H29A—C29—H29C	109.5
O11—C12—H12B	109.9	H29B—C29—H29C	109.5
C13—C12—H12B	109.9	O3—C30—O4	123.78 (11)
H12A—C12—H12B	108.3	O3—C30—C26	124.56 (11)
O14—C13—C12	106.22 (9)	O4—C30—C26	111.67 (10)
O14—C13—H13A	110.5	O4—C31—H31A	109.5
C12—C13—H13A	110.5	O4—C31—H31B	109.5
O14—C13—H13B	110.5	H31A—C31—H31B	109.5
C12—C13—H13B	110.5	O4—C31—H31C	109.5
H13A—C13—H13B	108.7	H31A—C31—H31C	109.5
C15—O14—C13	118.32 (9)	H31B—C31—H31C	109.5
O14—C15—C16	123.61 (10)	C22—C32—H32A	109.5
O14—C15—C20	115.78 (10)	C22—C32—H32B	109.5
C16—C15—C20	120.60 (11)	H32A—C32—H32B	109.5
C17—C16—C15	119.83 (11)	C22—C32—H32C	109.5
C17—C16—H16	120.1	H32A—C32—H32C	109.5
C15—C16—H16	120.1	H32B—C32—H32C	109.5
C18—C17—C16	120.47 (11)	C24—C33—H33A	109.5
C18—C17—H17	119.8	C24—C33—H33B	109.5
C16—C17—H17	119.8	H33A—C33—H33B	109.5
C17—C18—C19	119.25 (11)	C24—C33—H33C	109.5
C17—C18—H18	120.4	H33A—C33—H33C	109.5
C19—C18—H18	120.4	H33B—C33—H33C	109.5
C20—C19—C18	121.80 (11)		
			
N25—C1—C2—C3	−124.90 (10)	N25—C21—C22—C23	−63.60 (10)
C24—C1—C2—C3	110.89 (11)	C20—C21—C22—C23	171.03 (9)
N25—C1—C2—C7	59.33 (13)	N25—C21—C22—C32	173.28 (9)
C24—C1—C2—C7	−64.88 (12)	C20—C21—C22—C32	47.91 (13)
C7—C2—C3—C4	0.94 (16)	C32—C22—C23—O5	4.30 (15)
C1—C2—C3—C4	−175.04 (10)	C21—C22—C23—O5	−119.16 (11)
C2—C3—C4—C5	−1.41 (17)	C32—C22—C23—C24	−177.93 (9)
C3—C4—C5—C6	0.54 (17)	C21—C22—C23—C24	58.60 (11)
C4—C5—C6—C7	0.76 (17)	O5—C23—C24—C33	2.98 (15)
C5—C6—C7—O8	178.88 (10)	C22—C23—C24—C33	−174.79 (9)
C5—C6—C7—C2	−1.24 (16)	O5—C23—C24—C1	126.69 (11)
C3—C2—C7—O8	−179.72 (9)	C22—C23—C24—C1	−51.08 (12)
C1—C2—C7—O8	−3.90 (15)	N25—C1—C24—C23	47.70 (11)
C3—C2—C7—C6	0.40 (15)	C2—C1—C24—C23	173.02 (8)
C1—C2—C7—C6	176.21 (10)	N25—C1—C24—C33	171.55 (8)
C6—C7—O8—C9	4.66 (15)	C2—C1—C24—C33	−63.13 (11)
C2—C7—O8—C9	−175.22 (9)	C2—C1—N25—C26	42.83 (12)
C7—O8—C9—C10	175.16 (9)	C24—C1—N25—C26	166.55 (9)
O8—C9—C10—O11	−69.40 (11)	C2—C1—N25—C21	178.07 (8)
C9—C10—O11—C12	162.34 (9)	C24—C1—N25—C21	−58.22 (11)
C10—O11—C12—C13	−152.82 (9)	C20—C21—N25—C26	−33.60 (13)
O11—C12—C13—O14	65.01 (12)	C22—C21—N25—C26	−159.34 (9)
C12—C13—O14—C15	179.28 (9)	C20—C21—N25—C1	−167.36 (9)
C13—O14—C15—C16	−4.05 (17)	C22—C21—N25—C1	66.89 (10)
C13—O14—C15—C20	176.40 (10)	C1—N25—C26—C27	−109.15 (11)
O14—C15—C16—C17	−178.01 (11)	C21—N25—C26—C27	117.77 (11)
C20—C15—C16—C17	1.53 (18)	C1—N25—C26—C30	69.80 (13)
C15—C16—C17—C18	0.1 (2)	C21—N25—C26—C30	−63.28 (13)
C16—C17—C18—C19	−1.3 (2)	N25—C26—C27—C28	172.93 (10)
C17—C18—C19—C20	0.99 (19)	C30—C26—C27—C28	−6.05 (17)
C18—C19—C20—C15	0.57 (18)	C29—O2—C28—O1	5.6 (2)
C18—C19—C20—C21	178.40 (11)	C29—O2—C28—C27	−179.12 (12)
O14—C15—C20—C19	177.74 (10)	C26—C27—C28—O1	−94.78 (16)
C16—C15—C20—C19	−1.83 (17)	C26—C27—C28—O2	89.87 (14)
O14—C15—C20—C21	0.00 (16)	C31—O4—C30—O3	−8.58 (18)
C16—C15—C20—C21	−179.57 (11)	C31—O4—C30—C26	171.27 (9)
C19—C20—C21—N25	121.39 (11)	C27—C26—C30—O3	5.07 (19)
C15—C20—C21—N25	−60.89 (14)	N25—C26—C30—O3	−173.87 (12)
C19—C20—C21—C22	−116.21 (12)	C27—C26—C30—O4	−174.78 (10)
C15—C20—C21—C22	61.51 (14)	N25—C26—C30—O4	6.28 (15)

### Hydrogen-bond geometry (Å, º)

**Table:**

*D*—H···*A*	*D*—H	H···*A*	*D*···*A*	*D*—H···*A*
C13—H13*A*···O1^i^	0.99	2.56	3.2877 (18)	130
C29—H29*A*···O3^ii^	0.98	2.44	3.2498 (18)	139
C33—H33*A*···O5^iii^	0.98	2.56	3.4092 (16)	145

Symmetry codes: (i) *x*−1, *y*, *z*; (ii) −*x*+2, −*y*, −*z*+1; (iii) −*x*+1, −*y*+1, −*z*.
